# Involvement of interaction between TRPM2 and IKCA1 in temperature-dependent movement and IL-1β production in mouse microglia

**DOI:** 10.1016/j.jphyss.2026.100077

**Published:** 2026-05-01

**Authors:** Aykut Deveci, Makiko Kashio, Sandra Derouiche, Jing Lei, Yuji Imaizumi, Susumu Ohya, Moe Iwata, Makoto Tominaga

**Affiliations:** a Division of Cell Signaling, National Institute for Physiological Sciences, National Institutes of Natural Sciences, Okazaki 444-8787, Japan; b Graduate Institute of Advanced Studies, The Graduate University for Advanced Studies (SOKENDAI), Okazaki 444-8585, Japan; c Department of Cell Physiology, Faculty of Life Sciences, Kumamoto University, Kumamoto 862-0973, Japan; d Department of Molecular & Cellular Pharmacology, Graduate School of Pharmaceutical Sciences, Nagoya City University, Nagoya 467-8603, Japan; e Department of Pharmacology, Graduate School of Medical Sciences, Nagoya City University, Nagoya 467-8601, Japan; f Thermal Biology Research Group, Nagoya Advanced Research and Development Center, Nagoya City University, Nagoya 467-8601, Japan

## Abstract

Transient potential melastatin 2 (TRPM2) plays important roles in Ca^2 +^ signaling in tissues and cells, and contributes to various cellular functions. The Ca^2+^ influx induced by TRPM2 may activate the intermediate conductance Ca^2+^-activated potassium channel (IKCA1/ K_Ca_3.1/ SK4) and trigger K^+^ efflux. Here we demonstrate that a functional interaction between TRPM2 and IKCA1 contributes to cell volume changes in HEK293T cells and mouse primary microglia. Ca^2+^ entering cells through TRPM2 causes K^+^ efflux, followed by cell shrinkage upon water efflux. In addition, mouse microglia exhibited temperature-dependent movement *in vitro* that was modulated by the TRPM2-IKCA1 interaction within the physiological body temperature range. This interaction was also found to be involved in cytokine production in microglia. Understanding how the TRPM2-IKCA1 interaction in microglia can promote cell movement and cytokine production could be valuable for developing new strategies for treatments of diseases involving TRPM2.

## Introduction

The divalent cation Ca^2+^ plays essential physiological roles in humans and animals by acting as an intracellular signalling messenger that controls diverse processes like proliferation, contraction and excitation in different cell types [1], [2]. Ca^2+^ homeostasis is crucial for cell survival [3]. To maintain this homeostasis, Ca^2+^ fluxes are mediated by transporters or ion channels located on the plasma membrane or intracellular compartment membranes [4]. Among these ion channels, transient receptor potential (TRP) channels that have high Ca^2+^ permeability serve as biosensors to mediate responses to various environmental conditions [5], [6]. The TRP channel TRP melastatin 2 (TRPM2) is a Ca^2+^-permeable, non-selective temperature-sensitive cation channel that is highly expressed not only in the brain, but also in other tissues such as the lung, liver, bone marrow, spleen and heart [7], [8] where it functions as a biosensor of various stimuli including oxidative stress. Additional roles for TRPM2 in the central nervous system (CNS) are related to its presence in microglia, which are host macrophages in the brain. TRPM2 in microglia appears to be responsible for pathophysiological activation through reactive oxygen species (ROS) and lipopolysaccharide (LPS)-mediated signalling [9]. However, most studies regarding TRPM2 function in the brain were conducted in the context of pathophysiological events of stroke/ischemia and neurodegeneration [10] where TRPM2 activation is associated with cytokine release, exacerbation of inflammation and initiation of neuronal death.

We previously reported that Ca^2+^ influx through several TRP channels that have high Ca^2+^ permeability interacts with the Ca^2+^-activated Cl^-^ channel anoctamin 1 (ANO1, also known as TMEM16A), which is activated by Ca^2+^ that enters cells through TRP channels. During Cl^-^ efflux through activated ANO1, cations, including K^+^, also move out of the cell and drive water efflux through water channels. However, which K^+^ channels are coupled with ANO1 is unknown. In addition, it is not known which kinds of K^+^ channels could be activated downstream of TRPM2 channel activation while TRPV4 is well studied for its interaction with small, intermediate and large conductance Ca^2+^-activated K^+^ channels which is involved in vascular functions either smooth muscle cells or endothelial cells [11], [12], [13], [14], [15], [16].

In this study, we focused on intermediate conductance of the Ca^2+^-activated potassium channel (IKCA1/ K_Ca_3.1/ SK4) as a K^+^ channel that could interact with TRPM2 since IKCA1 encoded by KCNN4 shares a common functional role with TRPM2 in terms of its activation in response to increased intracellular Ca^2+^ concentrations. This intrinsic feature allows IKCA1 channel to play key roles in controlling cellular excitability in neurons, as well as in maintaining K^+^ homeostasis and regulating cell volume in non-excitable cells [17]. IKCA1 channels are also responsible for activation and proliferation of smooth muscle cells and lymphocytes [18], [19]. In the CNS, IKCA1 channels are mainly localized in microglia and endothelial cells [20]. Microglial IKCA1 channels are reportedly participate in several cellular functions including respiratory burst, proliferation and migration as well as in LPS-mediated nitric oxide production [21], [22].

Microglia are a specialized population of non-excitable primary innate immune cells in the CNS [23] and are reported to express both of TRPM2 and IKCA1 channels [21], [24], which are involved in CNS homeostasis by monitoring changes in their microenvironment (resting state) or by adopting protective features (activated state) [25]. This phenotypic change in microglia confers motility to microglia and allows them to move actively by following a chemotactic gradient towards the injured site [26]. Previous studies established that microglial cells play an essential role in the aging brain and various CNS-related diseases (e.g. ischemic stroke, traumatic brain damage, Alzheimer’s disease (AD), Parkinson’s disease (PD), multiple sclerosis (MS), and amyotrophic lateral sclerosis (ALS)), by mediating neuroinflammation [27]. Dysregulation of neuron-microglia communication is one of the main causes of neurodegenerative diseases [28], [29]. In this study we demonstrated an interaction between TRPM2 and IKCA1 channels in both HEK293T cells and mouse brain microglia, and found that this interaction is involved in several microglia functions.

## Materials and methods

### Animals

C57BL/6NCr mice (SLC Japan) were used as the WT strain. TRPM2 KO mice were generously provided by Dr. Yasuo Mori (Kyoto University, Japan) [30]. For *in vitro* time-lapse imaging, postnatal pups (P0 through P3) were obtained from TRPM2KO genotype mice. Mice were housed in a controlled environment (12 h light/12 h dark cycle; room temperature, 22°C to 24°C; 50–60% relative humidity) with free access to food and water. All procedures involving the care and use of animals were approved by the Institutional Animal Care and Use Committee of the National Institutes of Natural Sciences and carried out in accordance with the NIH Guide for the care and use of laboratory animals (NIH publication no. 85–23, revised 1985).

### Cell culture and transfection

HEK293T cells were maintained in D-MEM (Wako) supplemented with 10% (volume/volume) fetal bovine serum (BioWest or Gibco), penicillin-streptomycin (50 units/mL and 50 μg/mL, respectively, Gibco) and GlutaMAX (2 mM, Gibco). Cells were seeded at densities of 5 × 10^5^ cells per 35 mm dish 24 h before transfection. For patch-clamp and cell volume change recordings, HEK293T cells cultured in OPTI-MEM I medium (Invitrogen) on 35 mm dishes were transfected with 0.7 μg expression vector and 0.1 μg pGreen-Lantern 1 cDNA (pGL1) using Lipofectamine (Invitrogen) and Plus reagents (Invitrogen) according to the manufacturer’s protocol. After incubation for 3 h to 5 h, cells were reseeded on cover glasses and further incubated at 37 °C in a humidified CO_2_ incubator. Cells were used for experiments 20 h to 26 h after transfection.

### Preparation of primary mouse microglia

Primary mouse microglia were obtained according to a previously described method (Doering 2010) with modification. Mixed glial cultures were prepared from cerebral hemispheres of postnatal (P0 through P3) pups. The tissues were minced with a sterile Pasteur pipette (IWAKI) and trypsinized (Gibco) for 8 min at 37 °C. Then DNase (Roche) was added and the suspension was immediately mixed before addition of horse serum (Biowest) and mixing. The cell suspension was centrifuged at 800 rpm for 6 min. The supernatant was withdrawn and the cells were resuspended in fresh medium and filtered through a 100 μm nylon cell strainer (Falcon). Dissociated and filtered cells were seeded as mixed glia cultures in 75 cm^2^ tissue flasks (Falcon) in D-MEM (Dulbecco’s Modified Eagle Medium-low glucose; Sigma-Aldrich) with 10% (volume/volume) heat-inactivated bovine serum (Sigma-Aldrich), penicillin-streptomycin (10 units/mL and 10 mg/mL, respectively, Gibco), bovine insulin (5μg/mL, Sigma-Aldrich), and glucose solution (2 mg/mL, Otsuka) and cultured for 10–20 days at 37 °C in a humidified CO_2_ incubator until the cells reached 100% confluency. Primary microglia were obtained by shaking the flasks containing the mixed glia cultures and collecting the supernatant with floating cells. Cells were seeded on 35 mm diameter dishes containing 12 mm cover glasses (Matsunami Glass, 1082511226 unwashed (hydrophilic)) at a density of 1 × 10^4^ cells/dish and incubated at 37 °C in a humidified CO_2_ incubator for patch clamp assays. For time-lapse imaging, cells were seeded on 35 mm diameter dishes with 14 mm glass bottoms (Matsunami Glass, D1153OH) at a density of 1 × 10^4^ cells/dish and incubated at 37 °C in a humidified CO_2_ incubator. Cells were seeded on 35 mm diameter dishes containing 27 mm cover glasses (Matsunami Glass, D1114OH) at a density of 2 × 10^6^ cells/dish and incubated at 37 °C in a humidified CO_2_ incubator for patch clamp assays. Cells were used for experiments within 6 days of isolation.

### Electrophysiology

Transfected HEK293T cells or primary mouse microglia on cover glasses were incubated in the culture medium at 37 °C. The cover glasses were washed with a standard bath solution containing 140 mM NaCl, 5 mM KCl, 2 mM MgCl_2_, 2 mM CaCl_2_, 10 mM HEPES, and 10 mM glucose adjusted to pH 7.4 with NaOH. The cover glasses were mounted in a chamber (Warner Instruments) connected to a gravity flow system to deliver various drug stimuli and bath solutions. Recording pipettes were fabricated with a horizontal puller (Sutter Instruments, Model P-97). For experiments shown in Fig. S1, we used KCl intracellular pipette solution containing 140 mM KCl, 10 mM HEPES, 5 mM EGTA and Ca^2+^ concentrations ranging from 0 to 10 μM at pH 7.4. For experiments shown in Fig. S2, we used KCl intracellular pipette solution containing 140 mM KCl,10 mM HEPES, 5 mM EGTA and 500 nM of Ca^2+^ at pH 7.4. For Fig. S3, the intracellular pipette solution contained 140 mM KCl, 10 mM HEPES, 5 mM EGTA and 100 μM ADPR. The extracellular solution was a standard bath solution. For experiments concerning cell volume changes, the KCl intracellular pipette solution described above was supplemented with 500 μM calcein (Sigma-Aldrich, C0875). For all experiments in which the intracellular free Ca^2+^ was fixed at 500 nM, 4.48 mM CaCl_2_ was added (calculated by Webmaxc, standard version) before adjusting the pH. For all experiments in which the intracellular free Ca^2+^ was fixed at 100 nM, 3.18 mM CaCl_2_ was added before the pH was adjusted. Data for analysis were sampled at 10 kHz and filtered at 5 kHz for whole-cell recording (Axon Digidata 1550 amplifier with pClamp software, Molecular Devices). Membrane potential was clamped at −60 mV for all cell line and mouse primary microglia measurements. Ramp pulses protocol from −100 to + 100 mV for 500 msec were applied every 5 s. All experiments were performed at room temperature.

### In-vitro time-lapse imaging of microglia movement

Glass bottom dishes containing primary mouse microglia were placed on the stage of a Fluoview FV1200 microscope (OLYMPUS) fitted with a temperature-controlled incubator (Tokai Hit) under a circulating mixture of gases (20% O_2_, 5% CO_2_, and 75% N_2_). The stage/cover temperature were set at 37 °C/40 °C (37 °C) or 40 °C /41 °C (40 °C) for each experiment at least 1 h before image acquisition. The temperature setting for each condition was determined in advance by measuring the medium temperature with a wire probe and a digital thermometer (Unique medial). Cells were imaged at 1 min intervals with a 20X phase contrast objective lens (Nikon Instech). Image acquisition was performed using Fluoview software with the Z-stack function. Focused images taken with the Z-stack function were automatically selected on ImageJ (NIH) in each frame correction and then reconstructed into a series of stacking images along the time axis. Microglia migration distances were analyzed using ImageJ with the Manual Tracking and Migration plugin tool. Excel software (Microsoft) was used to calculate the migration distances of individual cells. Data defining the migration distances of microglia were collected from at least four individual glass-bottom dishes and four different preparations by analyzing all cells or analyzing ≈ 30 randomly selected cells per dish. Statistical analysis for motility was performed using the Wilcoxon/Kruskal-Wallis test followed by the Steel-Dwass method with JMP 14 software. *P* values ≤ 0.05 were considered significant.

### Co-immunoprecipitation and western blotting

Based on experiments with different amounts of mouse TRPM2 and mouse IKCA1 plasmid DNA, we used 1 and 0.2 μg/μl plasmid DNA for TRPM2 and IKCA1, respectively, to transfect HEK293 cells on 35 mm dishes (Fig. S7). At 24 h after transfection, HEK293T cells were gently rinsed with cold 1x PBS and collected in 80 µL lysis buffer (PBS containing 1% Triton X-100, 1 mM phenylmethylsulfonyl fluoride, and 1 × protease inhibitor cocktail (Nacalai)). The resulting cell lysate was placed on ice for 15 min before centrifugation at 12,000 x g for 20 min. PNGase F (NEB, P0704)-treatment of cell lysates was conducted according to manufacturer’s direction except denature condition (37 °C, 1 h). All subsequent steps were performed at 4 °C. Recombinant Protein G agarose bead slurry (40 µL; Thermo Fisher Scientific, 15920010) was incubated with 0.5 μg rat anti-HA (Roche, 11867423001) for 4 h with agitation, and the antibody-bound resin was collected after three centrifuge and wash cycles using PBS containing 1% Triton X-100. Cell lysates were added to the resin and incubated overnight with agitation. Pull-down protein samples were eluted from the beads in 30 μL sample buffer containing 50 mM DTT by heating at 37 °C for 1 h following three centrifuge and wash cycles. Rabit anti-HA (MBL, 561) and rabbit anti-FLAG (MBL, PM020) antibodies were used to probe for TRPM2 and IKCA1, respectively.

### ELISA

Microglia were shaken from the co-culture layer and plated at 5 × 10^5^ cells per well in 24-well plates containing coated coverslips and microglia medium. The culture medium was changed 2 h later to 500 μL fresh microglial medium with 100 ng/mL LPS O26:B6 (Sigma-Aldrich), 10 μM TRAM-34 (Cayman Chemicals), or both) or without drugs. Cells were incubated for 48 h at 37 °C in a humidified 5% CO_2_ incubator. Supernatants were collected and used immediately for cytokine ELISA. The remaining samples were stored at −80 °C for future analysis. Mouse IL-1β was assayed using ELISA kits purchased from R&D systems (Minneapolis, MN) according to the manufacturer’s instructions. Plates were read with a Thermo Scientific Labsystems 1500 Multiskan. Statistical analysis of cytokine production was carried using a Wilcoxon/Kruskal-Wallis test followed by the Steel-Dwass method on JMP 14 software. p values ≤ 0.05 were considered significant.

### Chemicals

TRAM-34 (Cayman Chemicals) was dissolved in DMSO (Wako). ADPR (Sigma-Aldrich) was dissolved in Milli-Q water to make a 100 mM stock solution, which was aliquoted and stored at −80 °C for future experiments. LPS O26:B6 (Sigma-Aldrich) was diluted in Milli-Q water to make a 100 μg/mL stock solution, which was aliquoted and stored at −20 °C for future experiments. The aliquoted chemicals were diluted 1:1000 in the pipette solution patch-clamp (ADPR) or in the medium for ELISA and time-lapse imaging experiments. The final DMSO concentration did not exceed 0.1%.

## Statistical analysis

Data are represented as means ± SEM. The data distribution was first analyzed with the Shapiro-Wilk test. Statistical analysis was performed using Origin software (OrginLab) for Student’s *t*-test or ANOVA with post hoc Bonferroni test for parametric data. Non-parametric statistical analysis was performed on JMP 14 software using the Wilcoxon/Kruskal-Wallis test followed by the Steel-Dwass method. *p* values < 0.05 were considered significant.

## Results

### TRAM-34, a specific inhibitor of IKCA1 channel

1-[(2-Chlorophenyl)diphenylmethyl]-1H-pyrazole (TRAM-34) is a derivative of the azole antimycotic clotrimazole and has been shown to be a specific inhibitor of IKCA1 [31]. We tested the effect of TRAM-34 on mouse IKCA1 expressed in HEK293T cells. After activating IKCA1 channels with 500 nM Ca^2+^ in the pipette solution, which is closed to the EC_50_ values (Fig. S1), we applied 10 μM TRAM-34 extracellularly. IKCA1-mediated currents were abolished in the presence of TRAM-34 (Fig. S2), but mouse TRPM2 channels activated by 100 μM ADPR contained in the pipette solution were unaffected by TRAM-34 (Fig. S3). Meanwhile, IKCA1-mediated currents were not observed in the presence of ADPR and TRAM-34 in IKCA1-expressing HEK293T cells. Based on these results, TRAM-34 and ADPR can be used to discriminate TRPM2- and IKCA1-mediated current responses.

### Ca^2+^ influx through TRPM2 activated by ADPR causes IKCA1 activation in HEK293T cells

**Fig. 1:**
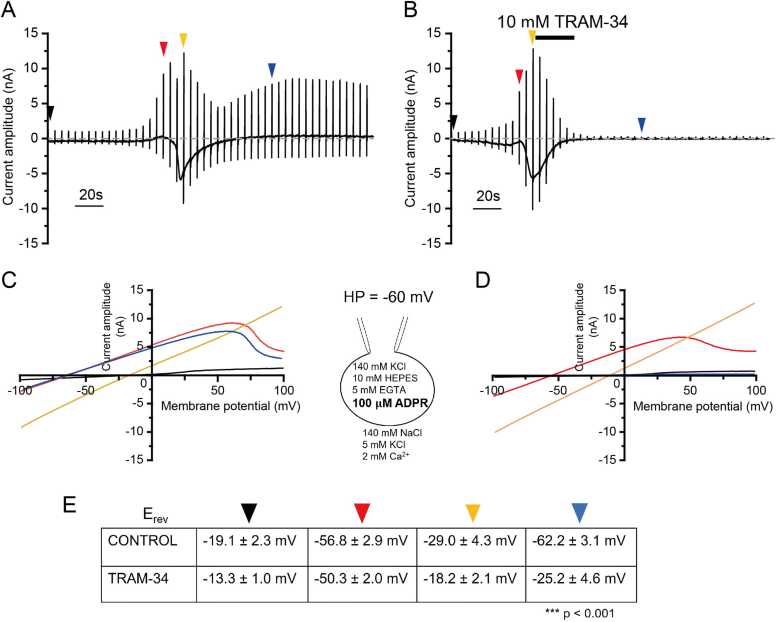
IKCA1 activation by Ca^2+^ entering HEK293T cells following ADPR induced-TRPM2 activation. A.B. Representative whole-cell current traces of HEK293T cells co-expressing IKCA1 and TRPM2. TRPM2 currents were induced with intracellular 100 μM ADPR without (A) or with (B) 10 μM TRAM-34. Ramp pulses from −100 mV to + 100 mV for 500 msec were applied every 5 s from the holding potential of −60 mV. C.D. Current-voltage (I-V) curves at time points indicated by the triangles in (A) and (B). E. Comparison of reversal potentials in the above experiments. Statistical significance (p < 0.001) was observed only between traces without and with TRAM-34. *** p < 0.001 (one-way ANOVA followed by post hoc Bonferroni test for multiple comparison).

In HEK293T cells co-expressing TRPM2 and IKCA1 channels, initial currents having reversal potentials (E_rev_) of −56.8 ± 2.9 mV (red arrowhead, n = 16) were followed by transient currents having a linear current-voltage (I-V) relationship when the pipette included ADPR (i.e., indicative of TRPM2-mediated currents), with an E_rev_ of −29.0 ± 4.3 mV (orange arrowhead, n = 16). Then, currents with E_rev_ of −62.2 ± 3.1 mV (blue arrowhead, n = 16) were slowly activated (Fig. 1 A, C, E); these currents were not observed in the presence of TRAM-34 (10 μM; n = 14; Fig. 1B, D, E). This shift in E_rev_ suggests that ADPR diffused into the patch-clamped cell and slowly activated TRPM2 that promoted both Na^+^ and Ca^2+^ influx that was followed by IKCA1 activation. The slowly activated IKCA1 channels with deep negative E_rev_ were completely inhibited by TRAM-34. The reported higher Ca^2+^ sensitivity of IKCA1 relative to TRPM2 [32], [33] could cause IKCA1 activation first. The fact that the E_rev_ of the currents with a linear I-V relationship was not close to 0 mV suggested simultaneous activation of TRPM2 and IKCA1 channels.

### IKCA1 activation induces cell shrinkage in HEK293T cells

**Fig. 2:**
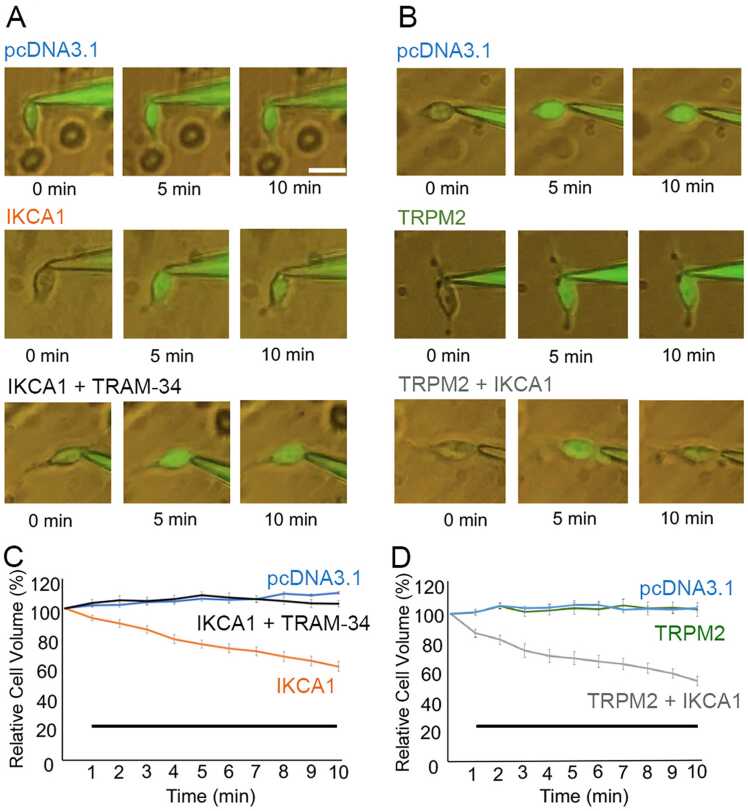
Effect of K^+^ flux on HEK293T cell volume changes. A.B. Representative cell volume changes for each condition. A. Cells expressing IKCA1 alone in the presence of 500 nM intracellular Ca^2+^ without (middle, orange, n = 14) and with (lower, red, n = 11) extracellular TRAM-34 compared with mock (pcDNA3.1)-transfected cells (upper, blue, n = 12). B. Cells expressing TRPM2 alone in the presence of 100 nM intracellular ADPR without (middle, green, n = 11) and with IKCA1 co-expression (lower, gray, n = 11) compared with mock-transfected cells (upper, blue, n = 11). The scale bar represents 20 μm. C.D. Plot of the relative percentage of cell volume throughout the recordings for each condition. Black bars indicate statistical difference of p < 0.001 between pcDNA3.1 and IKCA1 with TRAM-34 vs. IKCA1 without TRAM-34 (C) and between pcDNA3.1 and TRPM2 vs. TRPM2 with IKCA1 (D) (one-way ANOVA followed by post hoc Bonferroni test for multiple comparison).

An increase in K^+^ efflux through IKCA1 could be accompanied by water efflux that can cause cell shrinkage. We next examined cell volume changes during whole-cell patch-clamp experiments under different conditions. Cells could be clearly visualized with fluorescent calcein. We compared IKCA1-expressing cells and pcDNA3.1-transfected (mock) cells when the pipette solution contained 500 nM Ca^2+^. In a whole-cell configuration with cells expressing IKCA1 in the presence of intracellular 500 nM Ca^2+^, cells had shrunk by about 22% (22% ± 2%, n = 14) at 5 min and 35% (35% ± 3%, n = 14) at 10 min. This shrinkage was not seen for mock-transfected cells or IKCA1-expressing cells treated with 10 μM TRAM-34 (n = 11; Fig. 2 A, C), suggesting that IKCA1 channel activation by intracellular Ca^2+^ caused the cell shrinkage that may have been accompanied by water efflux.

In HEK293T cells expressing both TRPM2 and IKCA1 channels with intracellular ADPR that did not contain intracellular Ca^2+^, we observed shrinkage by 27% (27% ± 4%; n = 11) at 5 min and 40% (40% ± 3%; n = 11) at 10 min after making a whole-cell configuration in the presence of extracellular Ca^2+^. This result indicated that Ca^2+^ influx through ADPR-activated TRPM2 caused IKCA1 activation, followed by water efflux. Meanwhile, cells expressing TRPM2 alone had no shrinkage (n = 11; Fig. 2B, D). These results are consistent with previous studies showing that the K^+^ efflux is accompanied by Cl^-^ efflux and water efflux that decreases cell volume [34], [35].

### Physical interaction between TRPM2 and IKCA1 channels

**Fig. 3:**
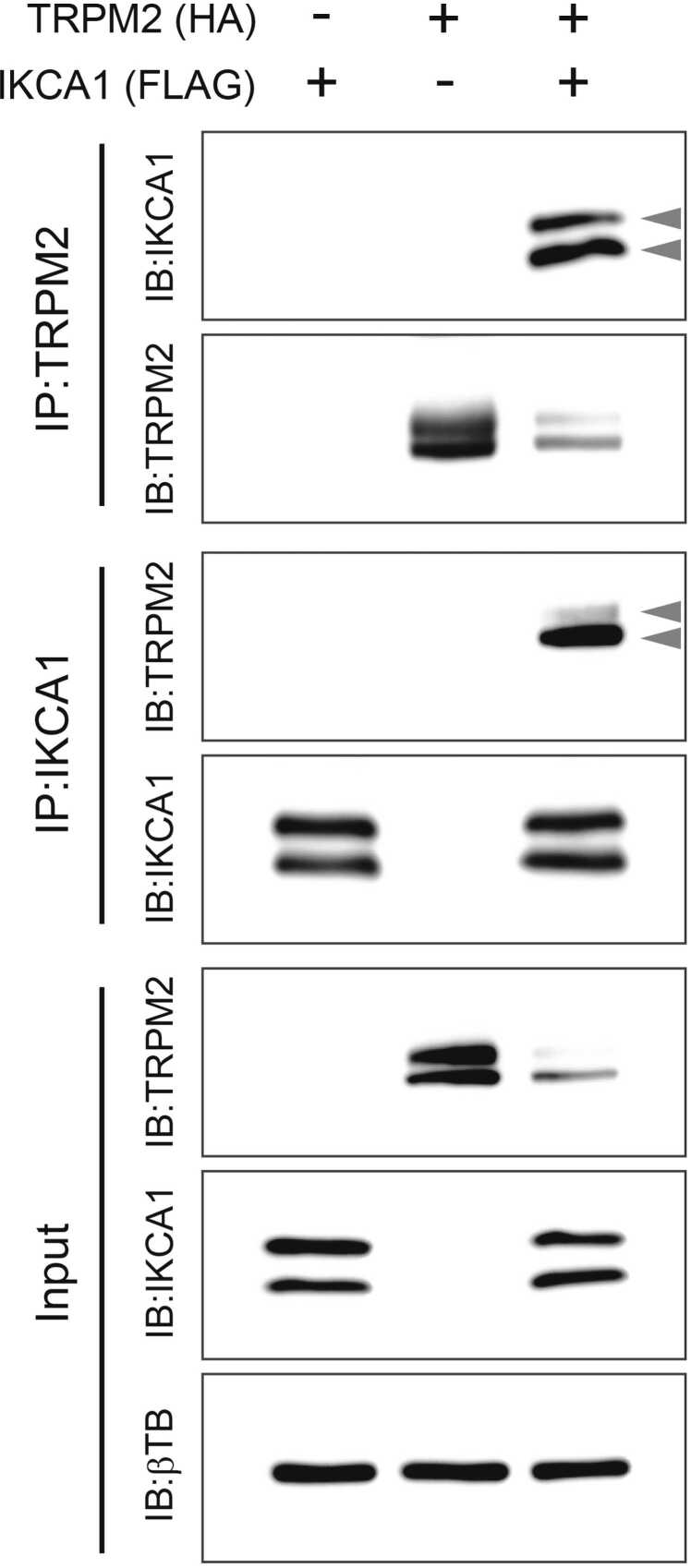
TRPM2 and IKCA1 form a physical complex. Co-immunoprecipitation (IP) of IKCA1 and TRPM2 in HEK293T cells. HEK293T cells were transiently transfected with empty vector, *HA-Trpm2* alone, *FLAG-IKCA* alone, or both *HA-Trpm2* and *FLAG-IKCA1* and were immunoprecipitated with anti-HA or anti-FLAG antibodies. Bands were detected using antibodies against the HA-tag and FLAG-tag. Selectively co-immunoprecipitated N-glycosylated (upper) and un-N-glycosylated (lower) forms of TRPM2 and IKCA1 are shown by arrowheads in the cells expressing both TRPM2 and IKCA1 (see Fig. S4-C). Input immunoblots indicate TRPM2, IKCA as well as β-tubulin (βTB) bands in the whole-cell lysates before co-IP. Original gel images are shown in Fig. S6.

Due to a lack of suitable antibodies for TRPM2 and IKCA1, we performed co-immunoprecipitation experiments using HA-tagged TRPM2 and FLAG-tagged IKCA1 with anti-HA and anti-FLAG antibodies, respectively, to examine whether TRPM2 and IKCA1 form a complex (Figs. S4, 5). The co-immunoprecipitation of TRPM2 and IKCA1 was observed only in lysates of HEK293T cells co-expressing TRPM2 and IKCA1 (Fig. 3, indicated by arrowheads) but not in HEK293T cells expressing TRPM2 or IKCA alone, clearly showing that TRPM2 indeed physically interacts with IKCA1 (Fig. 3 and Fig. S6). The upper bands of TRPM2 and IKCA1 are attributed to N-glycosylated forms, as they completely disappeared upon treatment with PNGase F cleaving N-linked glycosylation (Fig.S4-C).

### Activation of TRPM2 by ADPR (with 100 nM Ca^2+^) induces Ca^2+^ influx, followed by IKCA1 activation in mouse primary microglia

To reproduce the functional interaction between TRPM2 and IKCA1 channels observed in HEK293T cells in native cells, we chose mouse microglia, which are reported to functionally express both channels [21], [24], [29]. In addition, we previously showed that temperature-dependent microglia movement involves TRPM2 function [36]. We prepared nearly pure microglia cultures from newborn mouse brains (Fig. S7). The pipette solution used for channel measurements in mouse microglia contained 100 nM Ca^2+^ since IKCA1-mediated channel activation was not achieved with 100 μM ADPR alone (Fig. S8).

**Fig. 4:**
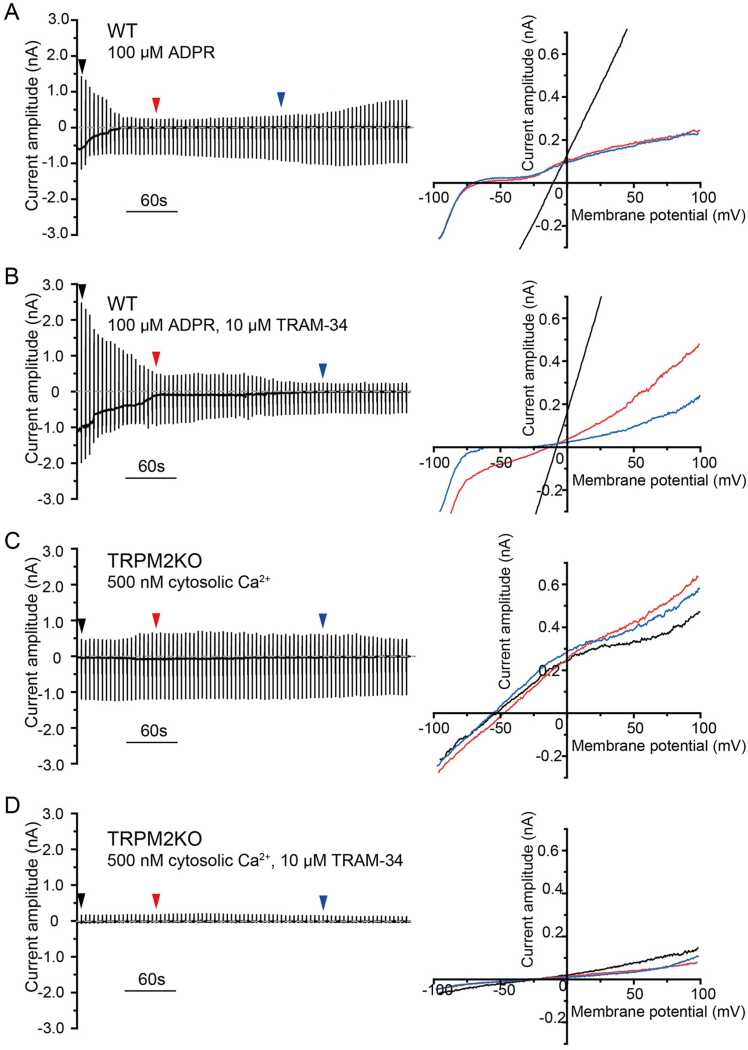
IKCA1 channel activation by Ca^2+^ entering mouse primary microglia through TRPM2 channels activated by ADPR. Representative whole-cell current traces of WT and TRPM2 KO mouse primary microglia with I-V curves at time points shown by colored triangles. Ramp pulses from −100 mV to + 100 mV for 500 msec were applied from the holding potential of −60 mV every 5 s. WT microglia with pipette solution containing 100 μM ADPR (n = 20) (A) or 100 μM ADPR in the presence of extracellular 10 μM TRAM-34 (B) (n = 12). TRPM2KO microglia with pipette solution containing (C) 500 nM Ca^2+^ (n = 11) or (D) 500 nM Ca^2+^ with 10 μM extracellular TRAM-34 (n = 12).

Wild-type (WT) microglia exhibited large TRPM2-mediated currents (E_rev_ of −17.2 ± 2.3 mV, n = 11) followed by IKCA1-mediated currents (E_rev_ of −56.1 ± 2.2 mV, n = 11; Fig. 4 A). The shift in E_rev_ was similar to that observed in HEK293T cells expressing both TRPM2 and IKCA1 channels. In the presence of TRAM-34 (10 μM; n = 12) the E_rev_ shift was abolished, although the initial ADPR-activated currents were still observed (Fig. 4B). Meanwhile, TRPM2KO cells exhibited neither TRPM2-mediated currents nor E_rev_ shift (Data not shown). TRPM2KO microglia did have clearly observable IKCA1-mediated currents (E_rev_ of −66.4 ± 3.1 mV, n = 11) in the presence of intracellular 500 nM Ca^2+^ (n = 12; Fig. 4C), but these currents disappeared when TRPM2KO microglia were treated with 10 μM TRAM-34 (Fig. 4D). Taken together, these results indicated that Ca^2+^ influx through TRPM2 activates IKCA1 in mouse microglia.

### Volume changes of mouse primary microglia depend on the TRPM2-IKCA1 interaction

**Fig. 5:**
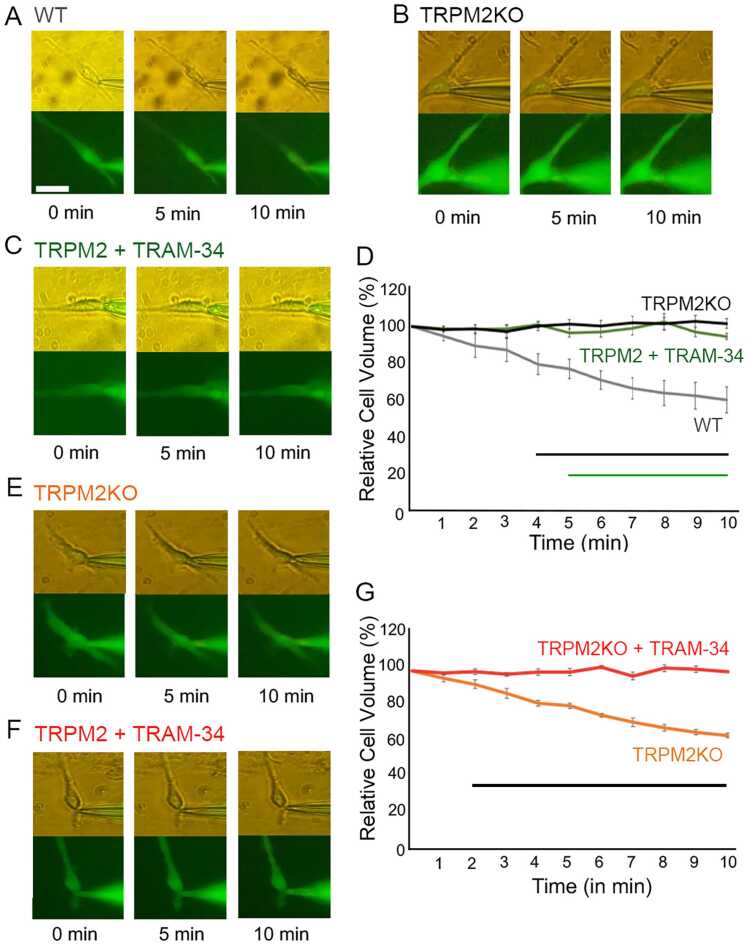
Effect of K^+^ flux on volume changes in mouse primary microglia. A.B.C.E.F. Representative cell volume changes in each condition with 100 nM intracellular Ca^2+^. A. WT microglia with KCl pipette solution containing 100 μM ADPR (gray, n = 5). B. TRPM2KO microglia with KCl pipette solution containing 100 μM ADPR (black, n = 5). C. WT microglia with KCl pipette solution containing 100 μM ADPR in the presence extracellular 10 μM TRAM-34 (green, n = 8). E. TRPM2KO microglia with KCl pipette solution containing 500 nM Ca^2+^ (orange, n = 6). F. TRPM2KO microglia with KCl pipette solution containing 500 nM Ca^2+^ in the presence of 10 μM extracellular TRAM-34 (red, n = 8). Scale bar represents 20 μm. (D) Plot of the percentage of relative cell volume changes from conditions in A (gray, n = 5), B (black, n = 5) and C (green, n = 8) and (G) from conditions in E (orange, n = 6) and F (red, n = 8). Black and green bars indicate statistical difference of p < 0.01 (green) or p < 0.001 (black) between TRPM2KO and WT or TRPN2 + TRAM34 and WT, respectively (D), and between TRPM2KO + TRAM-34 and TRPM2KO (G). One-way ANOVA followed by post hoc Bonferroni test was used for multiple comparisons.

We next used video recording during patch-clamp experiments to assess volume changes in mouse primary microglia (Fig. 5). First, we compared WT and TRPM2KO microglia activated by 100 μM ADPR in the pipette solution. Upon activation of TRPM2 by ADPR, WT primary mouse microglia shrank significantly by around 21% (21% ± 5%, n = 5) at 5 min and 36% (36% ± 6%, n = 5) at 10 min after making a whole-cell configuration (Fig. 5 A, D). This shrinkage was not seen for TRPM2KO primary microglia (Fig. 5B, D). Shrinkage of WT primary mouse microglia was abolished at 5 min and 10 min after making a whole-cell configuration when the cells were pre-treated with extracellular 10 μM TRAM-34 (Fig. 5C, D), indicating that cell volume changes were due to the activation of IKCA1 channels as was observed in HEK293T cells (Fig. 2). To confirm that the cell volume changes are indeed related to IKCA1 channel activation, we directly activated IKCA1 channels in TRPM2KO primary mouse microglia with 500 nM Ca^2+^ in the pipette solution. We observed significant cell shrinkage of around 20% (20% ± 1%, n = 6) at 5 min and 37% (37% ± 2%, n = 6) at 10 min after making a whole-cell configuration, and that this shrinkage was abolished in cells treated with TRAM-34 (Fig. 5E-G). These data indicated that in mouse primary microglia TRPM2 was activated by ADPR to allow Ca^2+^ to enter the cells. This Ca^2+^ entry activated IKCA1 channels to promote K^+^ efflux and in turn cell shrinkage.

### Microglia exhibit temperature- and TRPM2-IKCA1 interaction-dependent movement

**Fig. 6:**
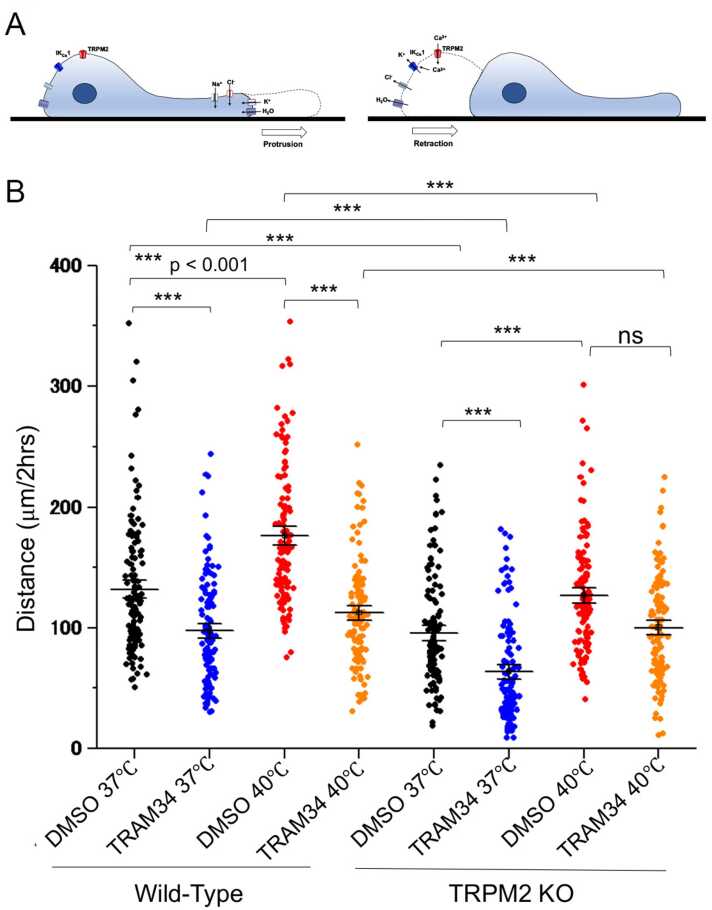
Effects of TRPM2-IKCA1 interaction on microglia movement. A. A model for microglia movement from the front (left) and tail (right). B. Average distances migrated by microglia isolated from WT, *Trpm2*-knockout (TRPM2KO) mice exposed to 37 °C or 40 °C in the presence of TRAM-34. Filled circles indicate migration distance by each microglial cell over the 2 h period. Horizontal lines indicate mean ± SEM. At 37 °C, WT and TRPM2KO microglia moved 132 μm ± 5 μm (n = 120) and 95 μm ± 4 μm (n = 120), respectively, and at 40 °C the microglia moved 176 μm ± 5 μm (n = 120) and 126 ± 4 μm (n = 120), respectively, in the absence of TRAM-34. In the presence of TRAM-34, WT and TRPM2KO microglia moved 97 μm ± 4 μm (n = 120) and 63 μm ± 4 μm (n = 120), respectively, at 37 °C, and 112 μm ± 4 μm (n = 120) and 100 μm ± 4 μm (n = 120), respectively, at 40 °C. *** p < 0.001 (two-way ANOVA followed by *post hoc* Bonferroni tests for multiple comparisons).

The volume changes observed in both HEK293T cells and mouse microglia prompted us to examine whether these channels are linked to cell migration. Since cell migration can be described as a repetitive cycle of protrusion of the cell front that is followed by retraction of the cell tail, the cycle can be modelled as volume gain at the cell front and volume loss at the tail of migrating cells [26]. Cell volume could increase during lamellipodium protrusion and decrease during tail retraction (Fig. 6A) [26]. Furthermore, local volume decreases of up to ∼35% have previously been visualized in migrating cells [37], [38]. These previously reported values are similar to those we observed in this study (Fig. 5).

To test our model, we carried out time-lapse imaging on WT and TRPM2KO microglia at 37 °C and 40 °C (Suppl. Video 1). The distance traveled by both WT and TRPM2KO microglia was significantly larger at 40 °C (176.1 ± 5.1 μm, n = 120 cells) than at 37 °C (131.9 ± 5.1 μm, n = 120 cells). At both temperatures, TRPM2KO migrated a significantly shorter distance than WT microglia (Fig. 6B), meaning that temperature-dependent movement of microglia involves TRPM2 as previously reported [36]. The temperature-dependent movement of TRPM2KO microglia was almost completely lost when cells were pre-treated with 10 μM TRAM-34 both at 37 °C (97.3 ± 4.1 μm, n = 120 cells) and 40 °C (112.4 ± 4.0 μm, n = 120 cells; Fig. 6B). All these data suggested that the TRPM2-IKCA1 interaction contributes to temperature-dependent mouse primary microglia movement and that migration is impaired when one of these two channels is deleted or inhibited.

Supplementary material related to this article can be found online at doi:10.1016/j.jphyss.2026.100077.

The following is the Supplementary material related to this article Video S1.

Video S1

### TRAM-34 treatment reduces IL-1β cytokine production in WT and TRPM2 KO mouse primary microglia

**Fig. 7:**
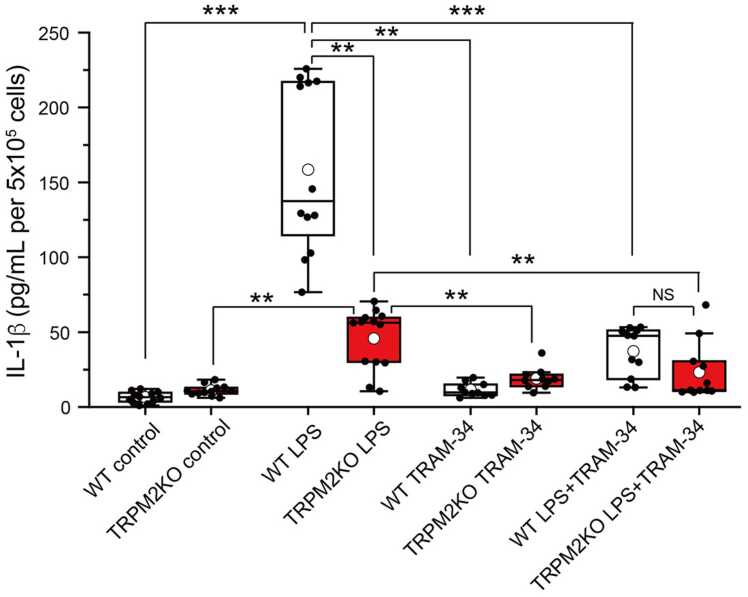
Effects of TRPM2-IKCA1 interaction on IL-1β production in mouse microglia IL-1β production in the absence and presence of LPS (100 ng/mL) in WT and TRPM2KO microglia in the presence and absence of TRAM-34. Box and whisker plots with mean (open circles) and individual data (closed circles; n = 12). ** p < 0.01, *** p < 0.001 (two-way ANOVA followed by *post hoc* Bonferroni tests for multiple comparisons).

Previous studies showed that both TRPM2 and IKCA1 channels individually contribute to IL-1β production [39], but whether the TRPM2-IKCA1 interaction plays a role in cytokine production in microglia is not known. Here we investigated whether the TRPM2-IKCA1 interaction contributes to IL-1β production in mouse primary microglia (Fig. 7). Stimulation of microglia with 100 ng/mL LPS induced a drastic increase in IL-1β release into the culture medium compared to the untreated control cells. A significant, but less prominent relative to WT, increase in IL-1β release was also observed in TRPM2KO microglia stimulated with 100 ng/mL LPS. These data suggested that TRPM2 is involved in IL-1β production in mouse microglia as previously reported [39]. To investigate whether IKCA1 channels are also involved in IL-1β production, WT and TRPM2KO microglia were stimulated with 100 ng/mL LPS in the presence of 10 μM TRAM-34. In WT and TRPM2KO mouse primary microglia, LPS-induced IL-1β production was almost completely abolished when IKCA1 channels were blocked by TRAM-34. Together, these data suggested that LPS-induced IL-1β production requires a TRPM2-IKCA1 interaction in mouse primary microglia.

## Discussion

TRPM2 and IKCA1 channels are widely studied individually, but their interaction has not been previously examined. In this study, we showed that Ca^2+^ entering cells through ADPR-activated TRPM2 activates IKCA1, causing K^+^ efflux that in turn drives water efflux. In HEK293T cells this outward water movement was associated with a 35–40% cell shrinkage, which is similar to the volume loss reported for IKCA1 activation that is followed by Cl^-^ and water efflux [34], [35]. The functional interaction of TRPM2-IKCA1 was found to be based on a physical interaction demonstrated in co-immunoprecipitation experiments (Fig. 3). Together, our results suggest that Ca^2+^ entering cells through TPRM2 activates both Ca^2+^-activated Cl^-^ and K^+^ channels, driving water efflux that could involve a large complex with up to four channels (TRPM2, IKCA1, Ca^2+^-activated Cl^-^ channel and water channel).

We observed a functional interaction between TRPM2 and IKCA1 in HEK293T cells and primary mouse microglia, and a similar reduction in cell volume reduction (40% and 36% in HEK293T cells and mouse microglia, respectively). These data suggest that the interaction could play a role in a broad range of cell types that express both TRPM2 and IKCA1. Here, pipette solution containing 100 μM ADPR but no Ca^2+^ activated TRPM2 in mouse primary microglia, whereas IKCA1 activity was only observed when 100 nM Ca^2+^ was also present in the pipette solution. This result suggests that Ca^2+^ entering cells through TRPM2 was not sufficient to reach the Ca^2^ thresholds needed for IKCA1 activation (Fig. S8). Ca^2+^ influx through TRPM2 based on the resting intracellular Ca^2+^ (around 100 nM) could effectively activate IKCA1 in mouse microglia that likely express lower levels of IKCA1 channels compared to transfected HEK293T cells.

We also found that the functional interaction between TRPM2 and IKCA1 is involved in microglia movement. Cell migration is described as a repetitive volume gain in the front part of the cell and volume loss in the tail of the cell [26] (Fig. 6A), and the volume changes in these two regions should occur in concert. Accordingly, we interpreted that the observed cell shrinkage could explain the events occurring in the tail. If this is the case, there could be uneven distribution of TRPM2 and IKCA1 channel proteins between the front (lamellipodium) and the tail of the cell. Although precise visualization of TRPM2 distribution in the cell is difficult due to a lack of suitable antibodies, future studies could produce results to support this hypothesis. Previous studies visualized and quantified volume changes of up to 35% [37], [38], which were similar to the ∼37% cell shrinkage we saw for mouse primary microglia in the present study (Fig. 5) and supports the possibility that cell volume changes could indeed be linked to cell migration.

We previously reported that Ca^2+^ influx through TRPV3 promotes rapid wound healing through increased phosphorylation of EGF receptor in skin keratinocytes [40]. In our previous study with a conventional wound healing assay using a cell insert, we showed that Ca^2+^ entering human skin keratinocytes activates ANO1 to allow Cl^-^ influx that enhances cell proliferation by accelerating the cell cycle through reduction of the amount of phosphorylated p38 [40]. The finding that filling of the gap in the wound healing assay occurred over the course of several days supports the involvement of cell proliferation in healing. Therefore, we think that both a Ca^2+^-dependent increase in cell movement through EGF receptor phosphorylation and an increase in cell proliferation involving the interaction between TRPV3 and ANO1 could contribute to the apparent wound healing seen for skin keratinocytes. We also previously reported that TRPV4 and TRPM2 are involved in temperature-dependent microglia movement in mouse microglia cultures, with TRPV4 appearing to play a more prominent role than TRPM2 [36]. This possibility is reasonable since both TRPV4 and TRPM2 are activated by warm temperatures in the body temperature range of humans. Ca^2+^ entering microglia through TRPV4 and TRPM2 could contribute to cell movement in a different way. Ca^2+^ entering microglia through TRPV4 could enhance migration by promoting rearrangement of the actin cytoskeleton, whereas Ca^2+^ entering microglia through TRPM2 could contribute to cell migration through cell tail shrinkage. Cells like microglia that have a high capacity for movement could have multiple mechanisms that contribute to migration. The primary mechanism of microglia migration is unclear, but our results showing that inhibition of IKCA1 by TRAM-34 completely inhibited temperature-dependent microglia movement (Fig. 6) suggest that cell tail shrinkage is a main component of movement that involves stoppage of the extension-retraction cycle.

Both TRPM2 and IKCA1 are reported to play roles in cytokine production, and here we showed that the TRPM2-IKCA1 interaction in mouse microglia is also important for release of cytokines [22], [39], [40]. Interestingly, treatment of microglia with TRAM-34 almost completely abolished LPS-induced IL-1β production (Fig. 7), suggesting that the TRPM2-IKCA1 interaction is important for IL-1β production in mouse primary microglia. We speculate that the IL-1β production could occur when NLRP3 (NLR family pyrin domain containing 3) senses K^+^ efflux as was previously reported [41], [42]. The molecular mechanism by which NLRP3 is activated in response to K^+^ efflux is unclear, but NEK7 (NIMA-related kinase), which is reported to act as a NLRP3 binding-protein downstream of K^+^ efflux [41], [42] (Fig. S9), may play a role. Moreover, IKCA1 inhibition by TRAM-34 could lead to membrane depolarization of microglia which in turn decreases driving force of Ca^2+^ influx through TRPM2. Therefore, another possible regulation at IL-1β transcription level might be involved, because IL-1β mRNA expression is reportedly enhanced by Ca^2+^-dependent PKC activity in microglia [43]. And the idea that inhibition of IKCA-mediated depolarization by TRAM34 can reduce the driving for both TRPM2 and TRPV4 channels might explain the large reduction in the moving distance observed in microglia treated with TRAM-34 (Fig. 6).

To conclude, our study indicates that TRPM2 and IKCA1 interact and different microglial phenotypes are produced downstream of this interaction. As IKCA1 is reportedly upregulated in microglia under inflammation [21], the contribution of TRPM2-IKCA1 interaction could be larger in microglial functions of the inflammatory conditions. These phenotypes could provide insight for the development of treatments for CNS pathologies involving TRPM2 and IKCA1. Although interaction between TRPV4 and Ca^2+^-activated K^+^ channels has been well studied [11], [12], [13], [14], [15], [16], no other TRP channels expect for TRPM2 in this study were found as candidate molecules working with Ca^2+^-activated K^+^ channels while they also have high Ca^2+^-permeability [44]. Because we reported the interaction of ANO1 with TRPV4, TRPV3, TRPV1 and TRPA1 [34], [40], [45], [46], many other TRP channels with high Ca^2+^ permeability could interact with Ca^2+^-activated K^+^ channels and be involved in the various Ca^2+^-dependent cellular events.

## List of abbreviations

TRPM2, Transient potential melastatin 2; IKCA1, intermediate conductance Ca^2^^+^ -activated potassium channel; ANO1, anoctamin 1; CNS, central nervous system; ROS, reactive oxygen species; AD, Alzheimer’s disease; PD, Parkinson’s disease; MS, multiple sclerosis; ALS, amyotrophic lateral sclerosis; TRAM-34, Triarylmethane-34; ADPR, ADP-ribose.

## CRediT authorship contribution statement

**Moe Iwata:** Methodology. **Susumu Ohya:** Resources. **Yuji Imaizumi:** Resources. **Jing Lei:** Data curation. **Sandra Derouiche:** Data curation, Conceptualization. **Makiko Kashio:** Writing – original draft, Data curation, Conceptualization. **Aykut Deveci:** Writing – original draft, Data curation, Conceptualization. **Makoto Tominaga:** Writing – review & editing, Writing – original draft, Validation, Supervision, Funding acquisition, Conceptualization.

## Ethics approval and consent to participate

All the animal care and experimental procedures were approved by our Institutional Animal Care and Use Committee and followed the National Institutes of Health and National Institute for Physiological Sciences guidelines (21A008), and carried out in compliance with the ARRIVE guidelines.

## Declaration of Competing Interest

The authors declare the following financial interests/personal relationships which may be considered as potential competing interests: Makoto Tominaga reports financial support was provided by Japan Society for the Promotion of Science. If there are other authors, they declare that they have no known competing financial interests or personal relationships that could have appeared to influence the work reported in this paper.

## Data Availability

All data and materials used in the analysis are available in the manuscript and Supplementary Information. Source data are provided with this paper.

## References

[1] Pinto M.C. (2015). Calcium signaling and cell proliferation. Cell Signal.

[2] Munaron L., Antoniotti S., Lovisolo D. (2004). Intracellular calcium signals and control of cell proliferation: how many mechanisms?. Journal of Cellular and Molecular Medicine.

[3] Mincheva-Tasheva S. (2014). Apoptotic cell death and altered calcium homeostasis caused by frataxin depletion in dorsal root ganglia neurons can be prevented by BH4 domain of Bcl-xL protein. Human Molecular Genetics.

[4] Clapham D.E. (2007). Calcium signaling. Cell.

[5] Nilius B. (2007). Transient receptor potential cation channels in disease. Physiological Reviews.

[6] Kashio M., Tominaga M. (2022). TRP channels in thermosensation. Current Opinion in Neurobiology.

[7] Sumoza-Toledo A. (2011). Dendritic cell maturation and chemotaxis is regulated by TRPM2-mediated lysosomal Ca^2+^ release. FASEB journal: official publication of the Federation of American Societies for Experimental Biology.

[8] Kashio M. (2025). Thermosensitive TRPM2: the regulatory mechanisms of its temperature sensitivity and physiological functions. Journal of Physiological Sciences.

[9] Kraft R. (2004). Hydrogen peroxide and ADP-ribose induce TRPM2-mediated calcium influx and cation currents in microglia. American Journal of Physiology - Cell Physiology.

[10] Xie Y.F. (2011). Dependence of NMDA/GSK-3beta mediated metaplasticity on TRPM2 channels at hippocampal CA3-CA1 synapses. Molecular Brain.

[11] Sonkusare S.K. (2012). Elementary Ca^2+^ signals through endothelial TRPV4 channels regulate vascular function. Science.

[12] Zhang P. (2014). Nitric oxide and protein kinase G act on TRPC1 to inhibit 11,12-EET-induced vascular relaxation. Cardiovascular Research.

[13] Goedicke-Fritz S. (2015). Evidence for functional and dynamic microcompartmentation of Cav-1/TRPV4/K_Ca_ in caveolae of endothelial cells. European Journal of Cell Biology.

[14] Ma Y. (2015). Epoxyeicosatrienoic acids act through TRPV4-TRPC1-K_Ca_1.1 complex to induce smooth muscle membrane hyperpolarization and relaxation in human internal mammary arteries. Biochimica et Biophysica Acta.

[15] Chen Y.L. (2022). Novel smooth muscle Ca^2+^-signaling nanodomains in blood pressure regulation. Circulation.

[16] Mao A. (2023). Functional role of coupling the endothelial TRPV4 and K_Ca_3.1 channels in regulating coronary vascular tone. The British Journal of Pharmacology.

[17] Kshatri A.S., Gonzalez-Hernandez A., Giraldez T. (2018). Physiological roles and therapeutic potential of Ca^2+^ activated potassium channels in the nervous system. Frontiers in Molecular Neuroscience.

[18] Toyama K. (2008). The intermediate-conductance calcium-activated potassium channel K_Ca_3.1 contributes to atherogenesis in mice and humans. Journal of Clinical Investigation.

[19] Ohya S. (2011). Involvement of dominant-negative spliced variants of the intermediate conductance Ca^2+^-activated K^+^ channel, K_Ca_3.1, in immune function of lymphoid cells. Journal of Biological Chemistry.

[20] Pedarzani P., Stocker M. (2008). Molecular and cellular basis of small--and intermediate-conductance, calcium-activated potassium channel function in the brain. Cellular and Molecular Life Sciences.

[21] Bouhy D. (2011). Inhibition of the Ca^2+^-dependent K^+^ channel, KCNN4/K_Ca_3.1, improves tissue protection and locomotor recovery after spinal cord injury. Journal of Neuroscience.

[22] Nguyen H.M. (2017). Differential Kv1.3, K_Ca_3.1, and Kir2.1 expression in “classically” and “alternatively” activated microglia. Glia.

[23] Kettenmann H. (2011). Physiology of microglia. Physiological Reviews.

[24] Sita G. (2018). TRPM2 in the brain: role in health and disease. Cells.

[25] Wolf S.A., Boddeke H.W., Kettenmann H. (2017). Microglia in physiology and disease. Annual Review of Physiology.

[26] Schwab A. (2012). Role of ion channels and transporters in cell migration. Physiological Reviews.

[27] Wendimu M.Y., Hooks S.B. (2022). Microglia phenotypes in aging and neurodegenerative diseases. Cells.

[28] Sarlus H., Heneka M.T. (2017). Microglia in Alzheimer's disease. Journal of Clinical Investigation.

[29] Gao C. (2023). Microglia in neurodegenerative diseases: mechanism and potential therapeutic targets. Signal Transduction and Targeted Therapy.

[30] Yamamoto S. (2008). TRPM2-mediated Ca^2+^ influx induces chemokine production in monocytes that aggravates inflammatory neutrophil infiltration. Nature Medicine.

[31] Brown B.M., Pressley B., Wulff H. (2018). K_Ca_3.1 channel modulators as potential therapeutic compounds for glioblastoma. Current Neuropharmacology.

[32] Joiner W.J. (1997). hSK4, a member of a novel subfamily of calcium-activated potassium channels. Proceedings of the National Academy of Sciences of the United States of America.

[33] Ishii T.M. (1997). A human intermediate conductance calcium-activated potassium channel. Proceedings of the National Academy of Sciences of the United States of America.

[34] Takayama Y. (2014). Modulation of water efflux through functional interaction between TRPV4 and TMEM16A/anoctamin 1. FASEB Journal.

[35] Hayabuchi Y. (2017). The action of smooth muscle cell potassium channels in the pathology of pulmonary arterial hypertension. Pediatric Cardiology.

[36] Nishimoto R. (2021). Thermosensitive TRPV4 channels mediate temperature-dependent microglia movement. Proceedings of the National Academy of Sciences of the United States of America.

[37] Schneider S.W. (2000). Volume dynamics in migrating epithelial cells measured with atomic force microscopy. Pfluger Architects.

[38] Watkins S., Sontheimer H. (2011). Hydrodynamic cellular volume changes enable glioma cell invasion. Journal of Neuroscience.

[39] Kashio M. (2012). Redox signal-mediated sensitization of transient receptor potential melastatin 2 (TRPM2) to temperature affects macrophage functions. Proceedings of the National Academy of Sciences of the United States of America.

[40] Yamanoi Y. (2023). TRPV3-ANO1 interaction positively regulates wound healing in keratinocytes. Communications Biology.

[41] He Y., Hara H., Nunez G. (2016). Mechanism and regulation of NLRP3 inflammasome activation. Trends in Biochemical Sciences.

[42] He Y. (2016). NEK7 is an essential mediator of NLRP3 activation downstream of potassium efflux. Nature.

[43] Fu S.Y. (2019). PKC mediates LPS-induced IL-1beta expression and participates in the pro-inflammatory effect of A_2A_R under high glutamate concentrations in mouse microglia. Neurochemical Research.

[44] Gees M., Colsoul B., Nilius B. (2010). The role of transient receptor potential cation channels in Ca^2+^ signaling. Cold Spring Harbor Perspectives in Biology.

[45] Takayama Y. (2015). Pain-enhancing mechanism through interaction between TRPV1 and anoctamin 1 in sensory neurons. Proceedings of the National Academy of Sciences of the United States of America.

[46] Derouiche S. (2018). TRPV4 heats up ANO1-dependent exocrine gland fluid secretion. FASEB Journal.

