# Supplementary material for: Involvement of interaction between TRPM2 and IKCA1 in temperature-dependent movement and IL-1β production in mouse microglia
Source: J Physiol Sci. 2026 May 1;76(2):100077. doi: 10.1016/j.jphyss.2026.100077 (PMC13158756; doi:10.1016/j.jphyss.2026.100077)
Supplement: Supplementary file 2 — Supplementary material [file mmc1.docx]

**
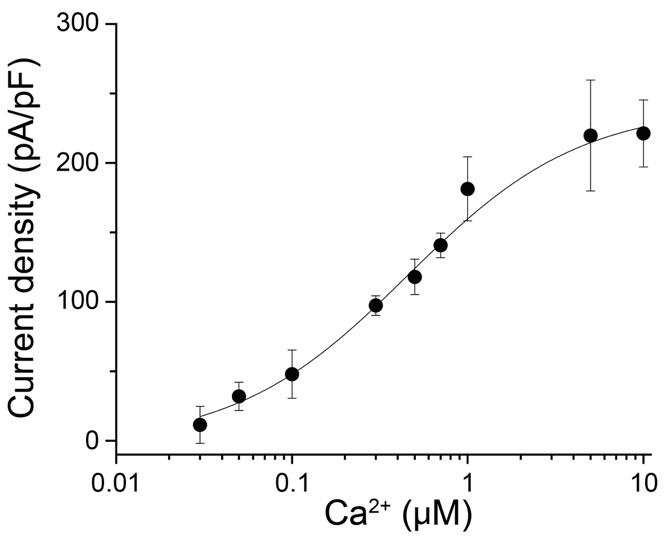
**

**Figure S1. Ca^2+^ dose-dependence of IKCA1 activation**

Ca^2+^ dose-dependency curve for IKCA1 activation. Data was fitted with the Hill equation and the calculated EC_50_ value was 450 nM (n = 5〜14).

**
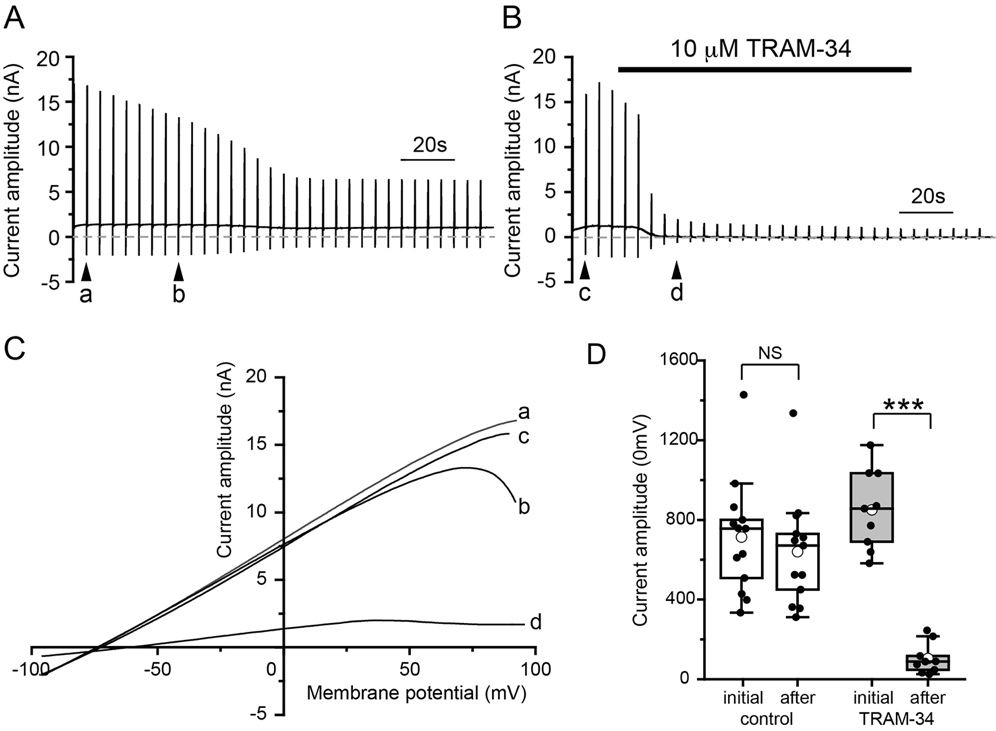
**

**Figure S2. Effect of TRAM-34 on IKCA1 channel activity**

**A.B.** Representative whole-cell current traces of TRPM2 channel activated by intracellular ADPR (100 μM) with ramp pulses from -100 mV to +100 mV over 500 msec every 5 sec from the holding potential of -60 mV in the absence (A) and presence (B) of TRAM-34 (10 μM; n=15 and 12, respectively). **C.** Current-voltage (I-V) curves at time points indicated by triangles. Peak current amplitudes of TRPM2 were 31.7 ± 7.4 nA and 28.0 ± 6.8 nA without and with TRAM-34 (10 μM), respectively. **D.** Transition of ADPR-evoked current amplitude (at 0mV) by vehicle (control) or TRAM-34 application in TRPM2/IKCA1-expressing cells. n=13 (control), n=9 (TRAM-34). ^NS^p>0.05, ***p<0.001 (one-way ANOVA followed by post hoc Bonferroni test for multiple comparison).

**
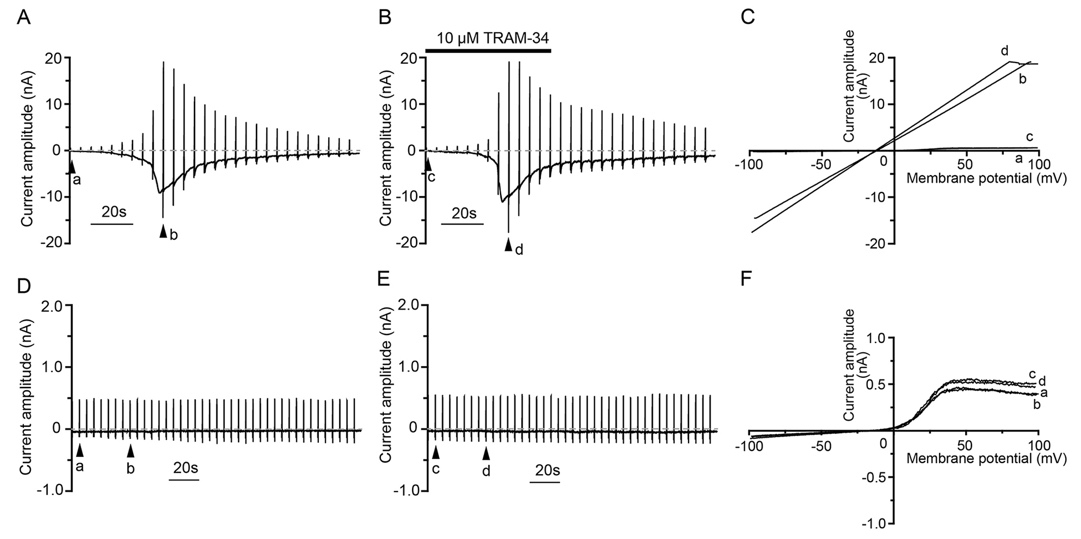
**

**Figure S3. Effects of TRAM-34 and ADPR on IKCA1 and TRPM2 channel activity**

**A.B.** Representative whole-cell current traces of TRPM2 channels activated by intracellular ADPR (100 μM) with ramp pulses from -100 mV to +100 mV over 500 msec every 5 sec from the holding potential of -60 mV in the absence (A) and presence (B) of TRAM-34 (10 μM; n=15 and 12, respectively). **C.** Current-voltage (I-V) curves at time points indicated by triangles. Peak TRPM2 current amplitudes were 31.7 ± 7.4 nA and 28.0 ± 6.8 nA without and with 10 μM TRAM-34 (10 μM), respectively. **D.E.** Representative whole-cell current traces of IKCA1-expressing cells in the presence of intracellular ADPR (100 μM) with ramp pulses from -100 mV to +100 mV over 500 msec every 5 sec from the holding potential of -60 mV in the absence (D) and presence (E) of TRAM-34 (10 μM; n=11 and 12). **F.** I-V curves at time points indicated by triangles.

**
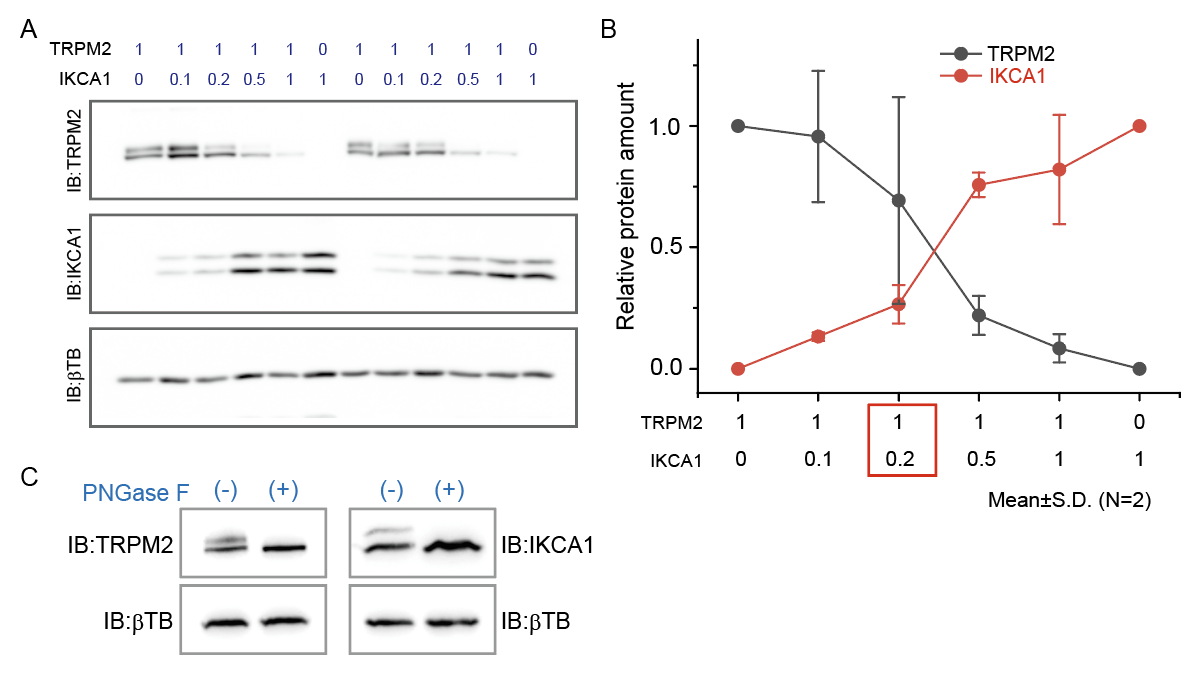
**

**Figure 4S. TRPM2 and IKCA1 protein expression in cells transfected with different amounts of TRPM2 and IKCA1 plasmid DNA**

**A.** TRPM2 and IKCA1 protein expression following transfection of HEK293T cells with different amounts of TRPM2 and IKCA1 plasmid DNA. **B.** Curves for relative amounts of TRPM2 (black) and IKCA1 (red) proteins upon transfection of HEK293T cells with different amounts of TRPM2 and IKCA1 plasmid DNA (n=2). Based on these results, 1 and 0.2 μg/μl plasmid DNA for TRPM2 and IKCA1, respectively, were used co-IP experiments. Original gel images are shown in Figure S5. C. The upper bands of both TRPM2 and IKCA1 disappeared upon PNGase F-treatment, confirming the presence of their N-glycosylated proteins

**Figure S5. Original gel images for Figure S4**

Cropped parts shown as dotted grey squares are used in Figure**
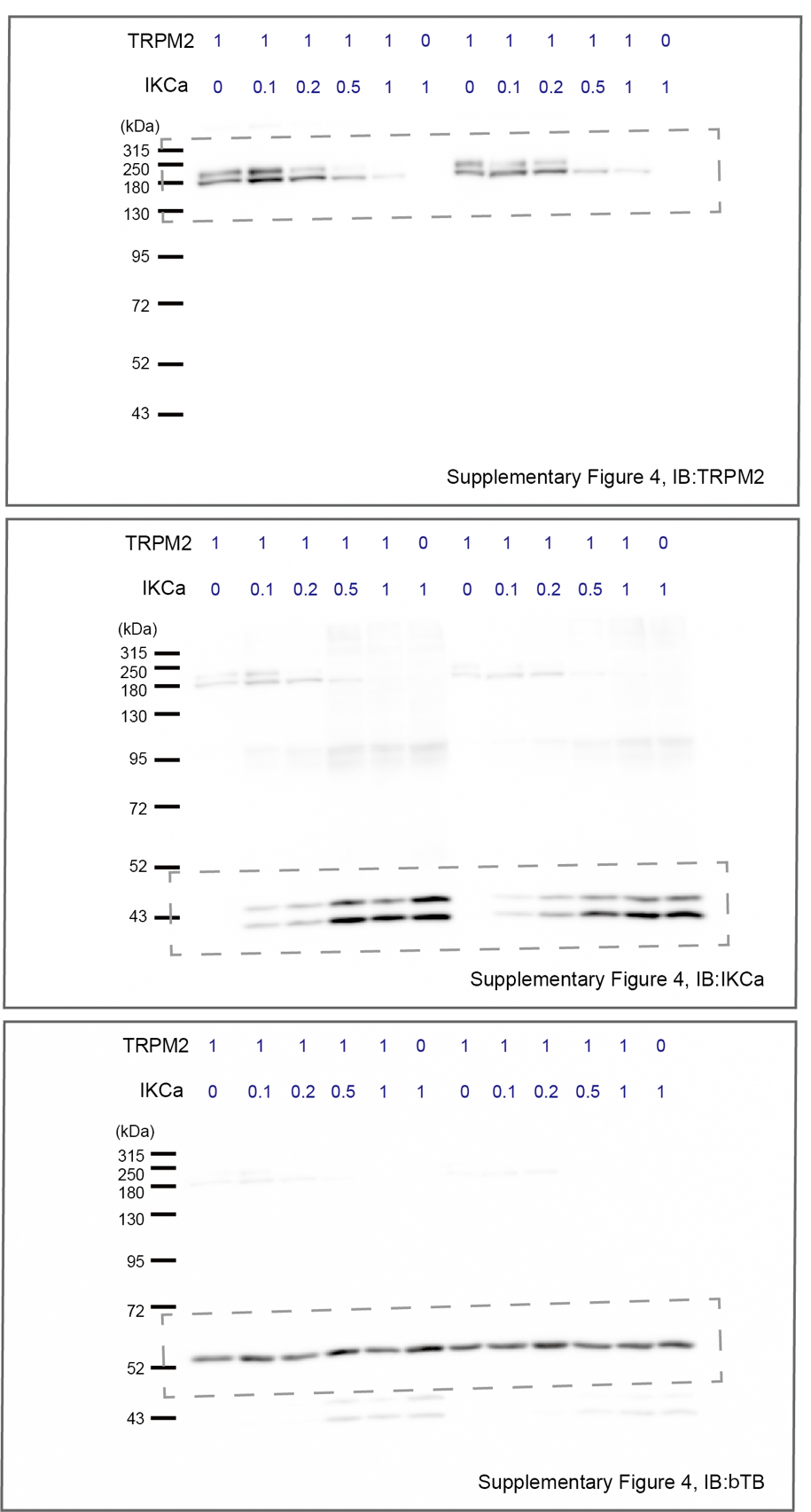
** S4.

**
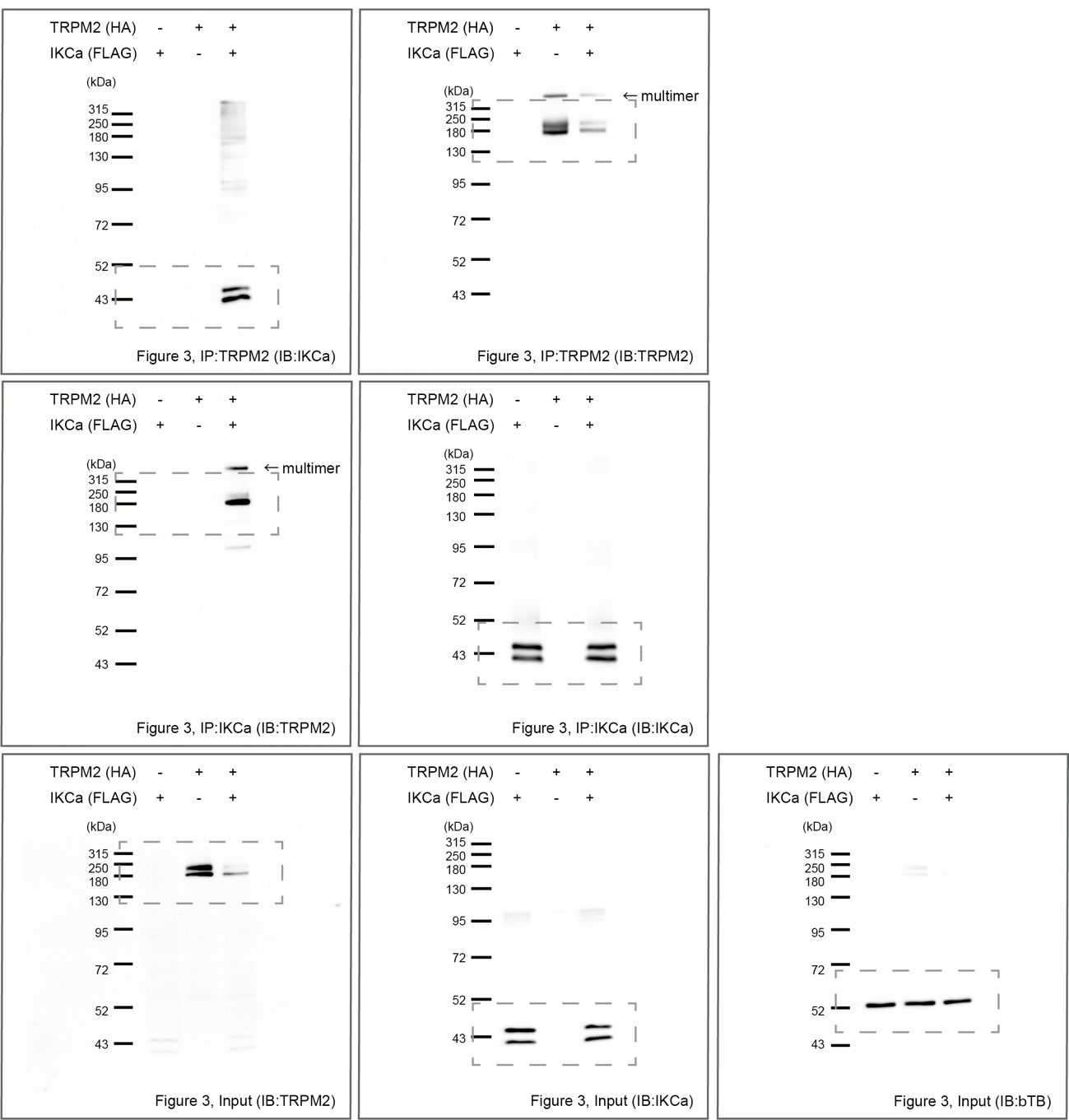
**

**Figure S6. Original gel images for Figure 3**

Cropped parts shown as dotted grey squares are used in Figure 3

**
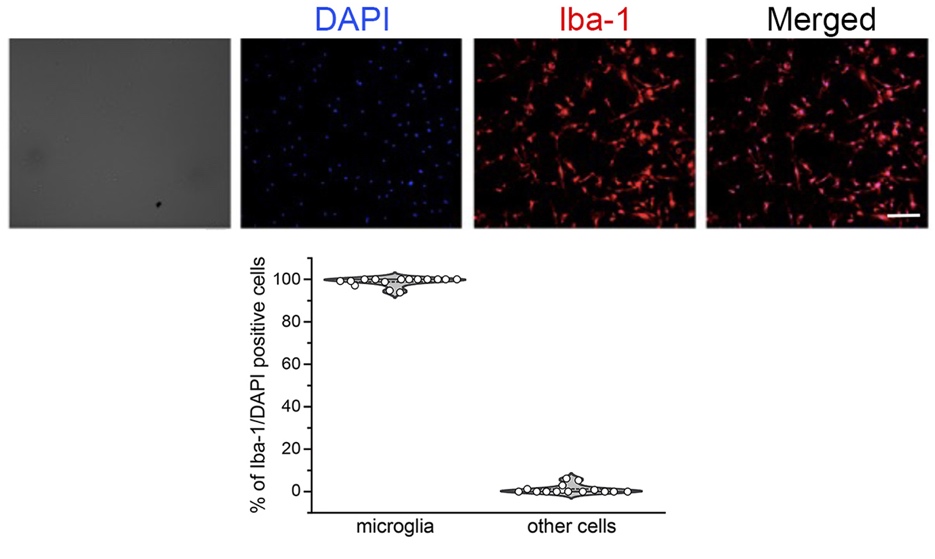
**

**Figure S7. P Purity of microglia isolated from mouse brain**

Representative image of microglia stained with an anti-Iba-1 antibody (from left to right: Bright field, DAPI, anti-Iba-1, Merged; Scale bars represent 100 μm) and violin plots for percentage of Iba-1/DAPI positive cells (~98%)

**
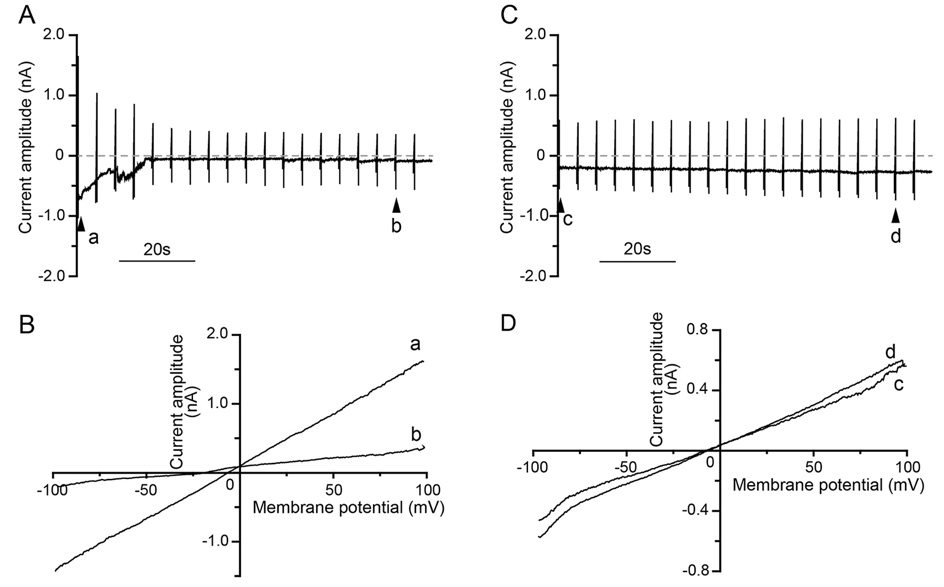
**

**Figure S8. Effect of intracellular Ca^2+^ on IKCA1 current activation**

**A.C.** Representative whole-cell current traces for WT (A, n = 17) and TRPM2KO (C, n = 6) mouse primary microglia with KCl pipette solution containing 100 μM ADPR in the absence of intracellular Ca^2+^. Ramp pulses from -100 mV to +100 mV over 500 msec every 5 sec from the holding potential of -60 mV. **B.D.** I-V curves at time points indicated in A (B) and C (D) by triangles.


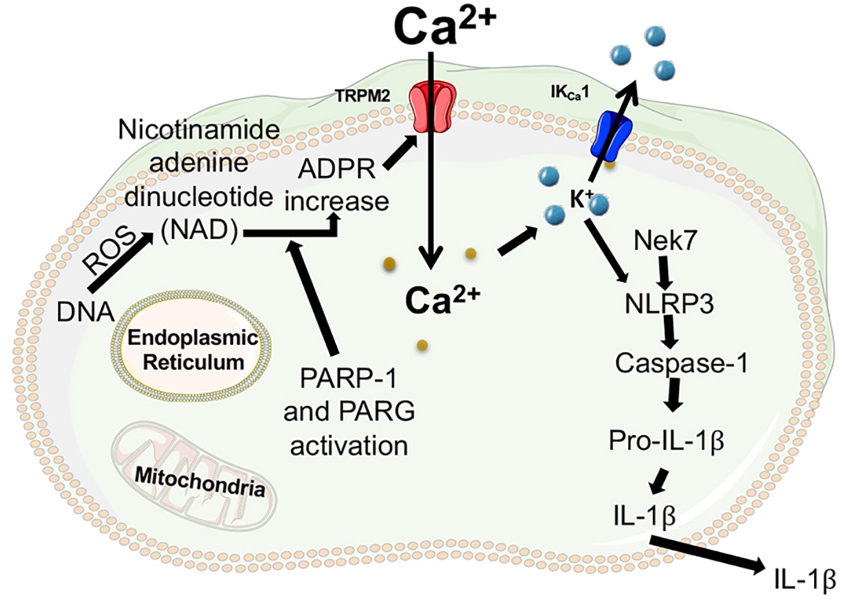


**Figure S9. Schematic model for IL-1β production in mouse microglia downstream of TRPM2 activation**

**Supplemental video 1. *In vitro* time-lapse imaging of WT and TRPM2 KO microglia expose to temperature changes and TRAM-34 treatment in relation to Figure 6**

Time-lapse imaging of WT and TRPM2 KO microglia was performed at different temperature (37 °C or 40 °C) in the presence or absence of TRAM-34 (Scale bar: 50 μm).
